# Cardioprotective Effect of the Compound *Yangshen* Granule in Rat Models with Acute Myocardial Infarction

**DOI:** 10.1155/2012/717123

**Published:** 2012-03-15

**Authors:** Xie Ming, Wang Tongshen, Wu Delin, Zhao Ronghua

**Affiliations:** ^1^Formulary Department, Beijing University of Chinese Medicine, Beijing 100029, China; ^2^Pharmacology Department, Anhui College of Chinese Medicine, Hefei, Anhui 230000, China

## Abstract

The protective effect of Compound *Yangshen* Granules was observed in myocardial infarction rat model. Rats were randomly divided into 6 groups: the model group, the control group (sham operated), the positive drug group, and small, medium, and large dosage of the *Yangshen* granule groups, respectively. The rats in the 3 *Yangshen* granule groups were orally administrated with 0.7 g/kg, 1.4 g/kg, and 2.8 g/kg for 7 consecutive days, whereas the rats of the positive drug group treated with 0.14 g/kg of Danshen Dropping Pills, and rats in the control and model groups orally administrated with saline. The rat model of acute myocardial infarction was established with ligation of coronary artery. Electrocardiograms at different time points, the blood rheology, myocardial enzymes, infarct size, and myocardial morphologic changes were measured. The results demonstrated that the granules could improve blood rheology, decrease st-segment of electrocardiograms and the activities of LDH and CK in serum, reduce myocardial infarction size, and alleviate myocardial histopathologic changes. In addition, the effect of the granules depended on the dose administrated orally. The results suggest that the *Yangshen* granules could produce cardioprotection effect and have potential benefits in the prevention of ischemic heart disease.

## 1. Introduction

Based on the principle of “nourishing the heart in summer” advocated by traditional Chinese medicine (TCM) and combined with the outlook that Chinese herbs and foods have the same source each other. Compound *Yangshen *Granule for summer health care is designed, which serves to clear the summer-heat, reinforce *qi* and nourish *yin* together protect the heart and regulate the mind as well. As a summer health formula of Chinese medicine, the granule is suitable for summer health care. In clinical, it was observed that *Shenmaiyin* which has the effect of reinforcing *qi* and nourishing *Yin* has the benefits for patients with coronary heart disease [1]. At the same time, experimental research has shown that *Shenmaiyin* have the cardioprotective effect of the rat model of acute myocardial infarction [2]. It suggested that formulas for reinforcing *qi* and nourishing *yin* may have the potential benefits in the prevention of coronary heart disease. This study is to provide the experimental evidence of the cardioprotective effect of the *Yangshen* granules in rat model of myocardial ischemia.

## 2. Materials and Methods

### 2.1. Animals

Sprague-Dawley (SD) rats (weight approximately 200–220 g) were purchased from Anhui Animal breeding laboratory of Animal Center, China (certificate no. SCXL (Anhui) 2005-001).

### 2.2. Herbs and Reagents

The formula of the Compound *Yangshen* Granule was provided by the Formula Laboratory of the Beijing University of Chinese Medicine (batch no: 20100202). The formula consisted of *ginseng *(Ren-Shen in Chinese), *Radix ophiopogonis* (Mai-Dong in Chinese), *Radix Puerariae *(Ge-Gen in Chinese), *Ziziphus jujuba seeds* (Suan-Zao-Ren in Chinese), *Japan-ese Honeysuckle *(Yin-Hua in Chinese), *Green tea* in proportions of 4 : 5 : 3 : 2 : 1 : 1. The processing flow of herbs is as follows: all the herbs were soaked in water for 2 hours, boiled and extracted for 3 times, 1 hour each time. Each time the proportions of water to herbs were 10 : 1, 8 : 1, and 8 : 1, and the decoction obtained was strained to put the filtrate together. After decompressed concentration (temperature ≤ 70°C) the relative density was 1.02~1.04 (60°C). Then 1% chitosan solution was added to be keept overnight and the solution was filtered. After decompressed concentration (temperature ≤ 70°C) to the relative density of 1.20~1.25 (60°C), the extracted liquid was dried (temperature ≤ 70°C) to get dry extract, which was crushed into fine dust and appropriate dextrin was added 90% ethanol was added to make granules go through a 14-meshed sift, and then dried (temperature ≤ 70°C) into granules through a 12-meshed sift. Each pouch contains 8.5 g granules (equal to 18 g crude drugs). Danshen Dropping Pills were produced by Tianjin Tasly Pharmaceutical Co, LTD (the product no. 20090722, Tianjing, China). The pills and Chinese granules were grinded and then mixed with distilled water before use. The kits of lactate dehydrogenase (LDH) and creatine kinase (CK) were provided by Nanjing Building Research Institute of Biological Engineering (the batch no. 201000118). TTC was purchased from Sigma (the batch no. T8877, USA).

### 2.3. Experimental Instruments

 The 754-A Spectrophotometer (Shanghai Scientific Instrument Company, Shanghai, China); Automatic Chemistry Analyzer (Olympus-400,USA); YDA-IV type machine of blood rheology (Beijing Hongrunda Sci-Tech Development Company, Beijing, China); XD-7100 Single Channel Electrocardiograph (Shanghai High-tech Medical Equipment Company, Shanghai, China).

### 2.4. Creation of the Acute Myocardial Ischemia Rat Model

The acute myocardial ischemia rat model was established according to [3–5]. Briefly, rats were given an operation in ether anesthesia and the chest was opened in the fourth left rib space to expose the heart. The left coronary arteries were then ligated with 5–0 lines from a distance of 2-3 mm to the root of the coronary artery.

### 2.5. Design and Allocation

The laboratory was set at 18°C and 55–65% humidity. Rats were randomly divided into 6 groups: the control group (sham operated), the model group, the positive drug group (Danshen Dropping Pill, DDP), and small, medium and large dose of *Yangshen* granule groups, 14 rats for each group. The rats in the small (SDG), medium (MDG) and large dose groups (LDG) were administrated orally with doses of 0.7 g/kg, 1.4 g/kg, and 2.80 g/kg (equivalent to 5, 10, and 20 times of an adult dosage of 8.5 g per day), respectively for 7 consecutive days. The rats in the positive drug group were treated with 0.14 g/kg of Danshen Dropping Pill, whereas the rats of the control and model groups were administrated orally with equivalent saline. 30 min after the last administration, rats were operated on by ligation of coronary artery. The electrocardiograms of each rat were recorded at different time points 2 h before and after operation. They were injected with 10% chloral hydrate after ligation for 24 h and the blood samples were taken from the abdominal aorta. After separation of serum and plasma (heparin anticoagulation), the relative myocardial enzymes and the blood viscosity were determined. After the heart was quickly removed, the weight of myocardial infarction was measured and myocardial pathology analyzed.

### 2.6. Detection of Different Markers

#### 2.6.1. Blood Rheology

 5 S^−1^, 30 S^−1^ and 200 S^−1^ of shear rate of the whole blood and the plasma viscosity were determined by a cone-plate-type Blood rheometer.

#### 2.6.2. Electrocardiogram (ECG)

Recorded by the II Guide League Electrocardiogram at the speed of 50 mm/s at different points 2 h after the ligation.

#### 2.6.3. Myocardial Infarction Size

 The heart was quickly removed from the body. After removal of the atrium and large blood vessels, the heart and ventricular weights were measured. The ventricule was horizontally cut into slices and incubated in 1% TTC solution in PBS at 37°C for 15 min. The red area indicated no myocardial infarction (mi), the area without color was the ischemic heart muscles. The weight of the ischemic heart muscle was measured and the rate of myocardial infarction calculated. Myocardial infarction rate (%) = infarction myocardial weight/the whole ventricular weight × 100%.

#### 2.6.4. CPK and LDH

 The abdominal aortic blood was separated by centrifugation at 3,000 rpm for 10 min. The activities of the serum enzymes were measured with the colorimetric method according to specifications of the kit.

#### 2.6.5. Myocardial Pathological Changes

 The cardiac muscles below ligation to the apex area of the heart was taken and fixed with a solution containing 10% formalin. The tissue slices of the heart were fabricated with HE dyeing. Histopathological changes were analyzed under a light-microscope and the severity was classified as described in references [6, 7].

### 2.7. Statistical Analysis

 All data were generated by the SPSS13.0 software. The data generated from multiple samples were statistically analyzed by One-way analysis of variance (ANOVA), SNK-Q tests, and a chi-square test. The data from the multiple samples for grade materials were analyzed by Kruskal-Wallis. A value of *P* < 0.05 was considered statistically significant.

## 3. Results

### 3.1. The Changes of Blood Rheology among Different Groups

Compared with the control group, the whole blood and plasma viscosities of the model group rats increase to different extent at different shear rates. It is significantly different (*P* < 0.05) at the high shear rate. Compared with the model group, the whole blood and plasma viscosities of the positive herbal group and different dose groups of granules decrease in different extents at different shear rates and those in the positive drug group and large-dose group of granule are significantly different (*P* < 0.05) at the high shear rate (see Table 1).

### 3.2. The Changes of St-Segment of Electrocardiograms among Different Groups

Compared with the control group, st-segment elevation of electrocardiograms at different time points are up in the model groups and the differences are all statistically significant (*P* < 0.05). Compared with the model group, st-segment elevation of electrocardiograms in the positive herbal group and different dose groups of granule reduces to different extent. The st-segment reductions of electrocardiograms are significantly different in the positive drug group at 0 and 0.5 h after surgery (*P* < 0.05). Those are also obviously significant in the small dose group of granule at 0 h and for the large dose group of granule at 0 h, 0.5 h, and 2 h after surgery (*P* < 0.05) (see Table 2).

### 3.3. The Changes of the Myocardial Infarction Rate among Different Groups

The average rate of myocardial infarction in the model group is 22.48%. Compared with model group, the average rates of the positive herbal group and different dose groups of granules descend to different extent. The differences were statistically significant in the positive herbal group and the medium and large dose groups of granules (*P* < 0.05) (see Table 3).

### 3.4. The Changes of Serum LDH and CK Activity among Different Groups

Compared with the control group, the serum LDH and CK activities in the model group increased significantly (*P* < 0.01) after coronary artery ligation for 24 h. Compared with the model group, the LDH and CK activities in the positive herbal group and different dose groups of granules decline to different extent. It is significantly different in the positive herbal group and the large dose group of granule (*P* < 0.05) (see Table 4).

### 3.5. The Changes of Myocardial Histopathology among Different Groups

**Figure 1:**
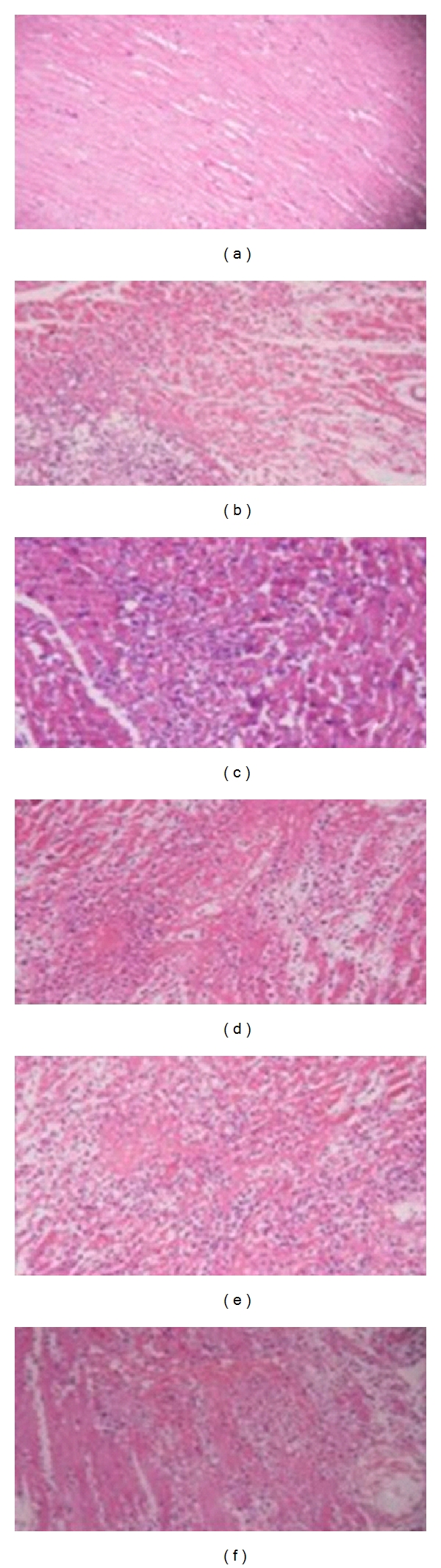
The myocardial protective effect of the Compound *Yangshen* Granule in the acute myocardium infarction rat model (HE, 10 × 10). (a) Control group; (b) model group; (c) positive dose group; (d) small dose group; (e) medium dose group; (f) large dose group.

The myocardial cells in the control group observed are arranged in order and cytoplasmic dyeing of the control group is of uniformity with the shape of round or oval. Meanwhile, nuclear chromatin in that group is uniformly distributed (see Figure 1(a)). Myocardial cells in the model group are dead with multifocal and flake coagulative characteristics. The nuclei are also dissolved and chipped. Some nuclear areas disappear and cytoplasm dyeing looks deeper. The blood vessels are of hyperemia obviously and mesenchymal edema with numerous neutrophile granulocytes infiltrating (see Figure 1(b)). The range of coagulative necrosis is smaller than the model group. The nuclei are dissolved and chipped in the infarction area. The nuclei in local area disappear and cytoplasm deeper dyeing. Some capillary congestion and mesenchymal edema with neutrophile granulocyte infiltrating are observed in the positive herbal group (see Figure 1(c)). The myocardial cells are partially in coagulative necrosis with globular or focal shape in various dose groups of granule. The myocardial degeneration appears but infarction is not obvious. In the infarction area, there are some cells with nucleus pyknosis and swelling or dissolved and chipped. Cytoplasm dyeing is deeper and the cell volume is decreased. The blood vessel congestion, edema, and neutrophil infiltration in cardiac interstitials are also observed. The pathological changes are markedly alleviated in the medium and large dose groups of granule compared with the model group (see Figures 1(d)–1(f)).

### 3.6. The Severity of Myocardial Damage among Different Groups

There are coagulative necrosis, interstitial hyperemia and bleeding, and inflammatory cells infiltrating in the heart tissues to different extent in various groups (*P* < 0.05). Compared with the model group, myocardial histopathologic changes decrease in the positive herbal group and the three dose groups of granule to different extent. The data are statistically significant in the positive herbal group and the medium and large dose groups of the granule (*P* < 0.05) (see Table 5).

## 4. Discussion

In summer, people tend to perspire more than other seasons, which may cause the imbalance of water, electrolyte, and the viscous blood. Meanwhile, the heart and kidney pressure increase and the physical function declines gradually, which possibly caused the aggravation of coronary heart disease. Based on the theory of TCM, it is well known that the heart connects correspond to fire, governs blood circulation and the mind as well. Meanwhile, summer connects correspond to fire and is also closely correlated with the heart. If the body is in disorder, it will cause deficiency of *qi *or *yin* due to hyperactivity of the heart, which results in various diseases. Keeping good health in summer should follow the principle of nourishing *qi *and *yin*, protecting heart, and regulating the mind. In the light of the experience of traditional Chinese medicine and according to the reference of guidebook for using herbs of health care products in Chinese homology of medicine and food [8], the formula of Compound *Yangshen* Granule for health care in summer is designed and it contains *Ginseng, Radix ophiopogonis, radix puerariae, Ziziphus jujuba seeds, Japan-ese Honeysuckle, green tea, *and so forth. This formula has potential effects such as clearing heat, or summer-heat, supplementing *qi *and nourishing *yin*, invigorating blood circulation and nourishing the heart.

Several studies have shown that *Ginseng* has obvious reaction to relieve stress [9]. It also has the protective effect for the myocardial cells with less oxygen and sugar [10] and can improve the blood rheology in the elderly [11]. *Radix ophiopogonis* can increase the tolerant ability of the body in hypoxia [12], repair ischemia myocardial, and improve the cardiac hemodynamic [13]. *Radix puerariae* may expand the coronary artery, protect the heart from myocardial ischemia, and improve the blood rheology [14, 15]. *Ziziphus jujuba seeds* can repair ischemia myocardial and reduce myocardial ischemic injury [16, 17].* Green tea* may alleviate fatigue [18] and has the effect of antioxidant [19]. These data suggest that this formula composed of the above Chinese herbal medicines may enhance the antistress ability and produce cardioprotection effects.

In this study, we know that the myocardial damage can be relieved in different dose groups, which prove that administration of the granules could improve the blood rheology, decrease st-segment of electrocardiograms, inhibit the activities of LDH and CK, reduce the myocardial infarction size, and alleviate the myocardial histopathologic changes in rat model. The observed effect of the *Yangshen* granules has highcorrelation with the doses of the granules treated for rats. It should be pointed out that administration of the granules at higher dose produces clear and significant effects of cardioprotection. Taken together, the data presented in this paper indicate that the granules have cardioprotection effects similar to other medicines for reinforcing *qi *and nourishing *yin* [1, 2]. These data also provide strong evidence implying that Compound *Yangshen* Granule may be used in the prevention of those patients with ischemic heart disease.

Danshen Dropping Pill has the effects of promoting blood circulation and removing obstruction of the vessels and relieving pain as well. It is an effective traditional Chinese patent medicine in the treatment of acute angina pectoris, and with the syndrome of *qi *and blood stasis in TCM. The *Yangshen* Granule acts to supplement *qi* and nourish *yin*, clear heat, and activate blood. It is designed for healthy population to keep good health under the summer heat or high temperature circumstance. It is especially for those with the syndrome of deficiency of both* qi *and *yin* due to summer heat. The two herbal medicines differ from each other and have their own indications. But the study has shown that both of them may have antimyocardial ischemia effect. It suggests that formulas with different treatments would have some identical pharmacological effects. However, the *Yangsheng *Granule and Danshen Dropping Pill are differed in composition of ingredients, indications, and administration in the light of the knowledge of TCM. It is necessary to have further study and evolution of their effects and characteristics.

## Figures and Tables

**Table 1:** The changes of blood rheology in the acute myocardial ischemia model treated by oral granules ($\overset{̅}{x}\pm s,\;n=10$, mPa/s).

Group	Dose (g*·*kg^−1^)		Whole blood viscosity		Plasma viscosity
		5 s^−1^	30 s^−1^	200 s^−1^	
Control	—	10.45 ± 0.43	6.88 ± 0.06	4.73 ± 0.03	1.59 ± 0.03
Model	—	10.99 ± 0.63	7.01 ± 0.13	4.84 ± 0.04**	1.62 ± 0.02
DDP	0.14	10.50 ± 0.26	6.91 ± 0.09	4.74 ± 0.06^##^	1.59 ± 0.02^#^
SDG	0.70	10.98 ± 0.37	6.99 ± 0.05	4.80 ± 0.03	1.61 ± 0.02
MDG	1.40	10.60 ± 0.72	6.92 ± 0.16	4.76 ± 0.08	1.60 ± 0.01
LDG	2.80	10.85 ± 0.52	6.95 ± 0.08	4.77 ± 0.07^##^	1.58 ± 0.02^#^

Note: **P < 0.01, compared with the positive drug group; ^#^ *P* < 0.05, ^##^ *P* < 0.01, compared with the model group.

**Table 2:** The changes of st-segment of electrocardiograms among different groups ($\overset{̅}{x}\pm s,\;n=10$, mV).

Group	Dose (g*·*kg^−1^)	St-segment elevation at different time after ligation			
		0 h	0.5 h	1 h	2 h
Control	—	0.15 ± 0.19	0.09 ± 0.12	0.09 ± 0.12	0.08 ± 0.09
Model	—	0.42 ± 0.17*	0.36 ± 0.17*	0.27 ± 0.11*	0.30 ± 0.16*
DDP	0.14	0.24 ± 0.11^#^	0.17 ± 0.11^#^	0.20 ± 0.12	0.19 ± 0.14
SDG	0.70	0.17 ± 0.09^#^	0.20 ± 0.19	0.21 ± 0.20	0.17 ± 0.12
MDG	1.40	0.27 ± 0.25	0.23 ± 0.19	0.18 ± 0.15	0.16 ± 0.14^#^
LDG	2.80	0.17 ± 0.14^#^	0.23 ± 0.10^#^	0.19 ± 0.16	0.14 ± 0.12^#^

Note: *P < 0.05, compared with the positive drug group; ^#^ *P* < 0.05, compared with the model group.

**Table 3:** The changes of myocardial infarction rate among different groups ($\overset{̅}{x}\pm s,\;n=10$, g).

Group	Dose (g*·*kg^−1^)	Weight of heart	Weight of ventricle	Weight of infarction	Rate of infarction
Control	—	0.8272 ± 0.1346	0.5678 ± 0.0995	—	—
Model	—	0.8964 ± 0.0960	0.6314 ± 0.0705	0.1440 ± 0.0519**	22.48 ± 6.77**
DDP	0.14	0.8132 ± 0.1183	0.5822 ± 0.0968	0.0411 ± 0.0188^#^	7.01 ± 1.49^##^
SDG	0.70	0.8906 ± 0.0825	0.6235 ± 0.0597	0.1016 ± 0.0307	16.05 ± 3.31
MDG	1.40	0.9097 ± 0.0841	0.6629 ± 0.0933	0.0895 ± 0.0413^#^	13.77 ± 7.18^#^
LDG	2.80	0.8194 ± 0.0525	0.5759 ± 0.0482	0.0574 ± 0.0173^##^	10.10 ± 3.61^##^

Note:**P < 0.01, compared with the positive drug group; ^#^ *P* < 0.05, ^##^ *P* < 0.01, compared with the model group.

**Table 4:** The changes of serum LDH and CK activities among different groups ($\overset{̅}{x}\pm s,\;n=10$).

Group	Dose (g*·*kg^−1^)	LDH (U/L)	CK (U/mL)
Control	—	1755.56 ± 667.22	0.25 ± 0.13
Model	—	3481.54 ± 569.16**	0.53 ± 0.11**
DDP	0.14	2321.63 ± 602.34^#^	0.27 ± 0.21^#^
SDG	0.70	3195.65 ± 739.48*	0.47 ± 0.19**
MDG	1.40	2940.32 ± 677.04*	0.40 ± 0.21
LDG	2.80	2619.14 ± 638.03^∗ #^	0.36 ± 0.13^#^

Note: *P < 0.05, **P < 0.01, compared with the positive drug group; ^#^ *P* < 0.05, compared with the control group.

**Table 5:** The myocardial histopathological changes among different groups.

Group	Dose (g*·*kg^−1^)	Early coagulation necrosis				Hyperemia bleeding				Inflammatory cells infiltrating			
		−	+	++	+++	−	+	++	+++	−	+	++	+++
Control	—	8	0	0	0	8	0	0	0	8	0	0	0
Model	—	0	1	6	1	0	0	7	1	0	4	4	0*
DDP	0.14	0	6	2	0	1	4	3	0	0	4	4	0^#^
SDG	0.70	0	1	7	0	0	2	5	1	0	4	4	0^#^
MDG	1.40	0	7	1	0	1	5	2	0	0	7	1	0^#^
LDG	2.80	2	5	1	0	0	6	2	0	2	6	0	0^#^

Note: *P < 0.05, compared with the positive drug group; ^#^ *P* < 0.05, compared with the control group.
